# Dysregulated Interferon Response Underlying Severe COVID-19

**DOI:** 10.3390/v12121433

**Published:** 2020-12-13

**Authors:** LeAnn Lopez, Peter C. Sang, Yun Tian, Yongming Sang

**Affiliations:** Department of Agricultural and Environmental Sciences, College of Agriculture, Tennessee State University, 3500 John A. Merritt Boulevard, Nashville, TN 37209, USA; lhellese@my.tnstate.edu (L.L.); peter.sangc@gmail.com (P.C.S.); ytian@tnstate.edu (Y.T.)

**Keywords:** COVID-19, interferons, interferon signaling, SARS-CoV-2, immunopathy

## Abstract

Innate immune interferons (IFNs), including type I and III IFNs, constitute critical antiviral mechanisms. Recent studies reveal that IFN dysregulation is key to determine COVID-19 pathogenesis. Effective IFN stimulation or prophylactic administration of IFNs at the early stage prior to severe COVID-19 may elicit an autonomous antiviral state, restrict the virus infection, and prevent COVID-19 progression. Inborn genetic flaws and autoreactive antibodies that block IFN response have been significantly associated with about 14% of patients with life-threatening COVID-19 pneumonia. In most severe COVID-19 patients without genetic errors in IFN-relevant gene loci, IFN dysregulation is progressively worsened and associated with the situation of pro-inflammation and immunopathy, which is prone to autoimmunity. In addition, the high correlation of severe COVID-19 with seniority, males, and individuals with pre-existing comorbidities will be plausibly explained by the coincidence of IFN aberrance in these situations. Collectively, current studies call for a better understanding of the IFN response regarding the spatiotemporal determination and subtype-specificity against SARS-CoV-2 infections, which are warranted to devise IFN-related prophylactics and therapies.

## 1. Diverted Type I Interferon (IFN) Response Associated with Hyper-Inflammation

**Figure 1:**
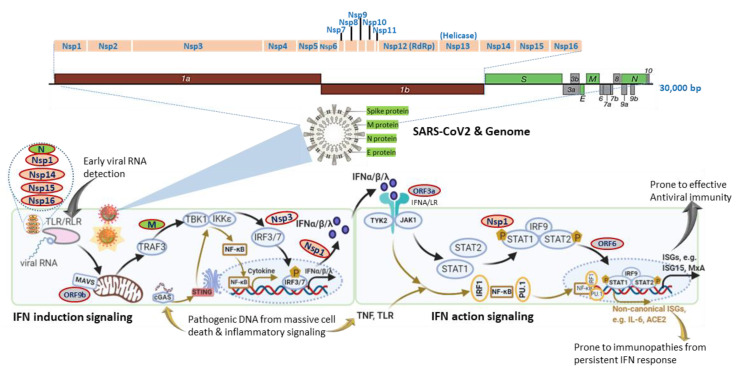
SARS-CoV-2 genomic structure and analogical antagonism to interferon (IFN) signaling. Analogical to typical human β-coronaviruses, the SARS-CoV-2 genome contains ORF1a/1b, encoding a polyprotein, which is proteolytically processed into non-structural protein (Nsp) 1–16 (top schematic). Structural proteins, including spike (S), envelope (E), membrane (M), and nucleocapsid (N) proteins, are diagramed to depict the genome and viron structures (middle). Other accessory proteins encoded at the 3′ end of the viral genome comprise ORF3a, 3b, 6, 7a, 7b, 8, 9a, 9b, and 10 (colored in grey). The bottom panel depicts SARS-CoV-2 proteins (colored ovals with red outlines) that interfere with either IFN induction or action pathways and are posited next to their known or hypothetic targets/steps in the IFN signaling. SARS-CoV-2 seems to evolve multiple antagonistic mechanisms against the host IFN signaling and especially those on early IFN induction signaling. Note, cellular IFN induction may go with either a MAVS- or STING-dependent pathways that respond to cytosolic pathogenic RNA or DNA molecular patterns, respectively. Similarly, IFN action signaling may lead through a canonical ISGs induction with limited pro-inflammation or crosstalk with inflammatory signaling from TNF and TLR to increase the expression of non-canonical ISGs accompanying a pro-inflammatory and autoimmune ambient through epigenetic regulation. The canonical IFN signaling flow, which acts generally at an early stage of SARS-CoV-2 infection for primarily restricting viral infection, is depicted using black arrows, and brown arrows represent the non-canonical IFN signaling flow activated at a later stage in severe COVID-19, which is highly associated with pro-inflammation and immunopathies.

The severe acute respiratory syndrome coronavirus 2 (SARS-CoV-2), which causes the current pandemic of new coronavirus disease 2019 (COVID-19), shows an evolutionary success to adapt its infectivity and contagiousness to efficiently spread in human societies [1,2,3,4,5,6]. The prognosis of SARS-CoV-2-infected patients is very broad, with a vast majority of people (50–80% based on different research scenarios, CDC) only having mild symptoms like the common cold or asymptomatic [7]; however, still, the other significant numbers (averagely 20–50% based on different ethnicity and pre-medical conditions) may progress into severe respiratory and systemic syndromes, needing immediate hospitalization and critical care [8,9,10,11,12]. The case fatality rate of COVID-19 ranges from 1.7–13.0% in different countries [7]. Except for the pathogenic impact of viral infection, major pathologies underlying severe COVID-19 come from the dysregulation of vast immune factors at both the cellular and molecular levels. For example, severe COVID-19 patients display macrophage overreaction (also known as macrophage activation syndrome (MAS)) and lymphopenias of effective lymphocytes, including neutrophils, CD4 T cells, and natural killer (NK) cells [13,14,15]. At the molecular level, hyper-regulation of pro-inflammatory mediators (including IL-6, TNFα, S100A8/9, and C-reactive protein), a significant decrease of human leukocyte antigen D-related (HLA-DR) gene expression in CD14 monocytes, and dysregulated antiviral interferon (IFN) response have been reported in COVID-19 patients with critical illness [13,14,15]. In this review, we focus on the determinant role of dysfunctional IFN response underlying the progression of severe COVID-19. Interferon (IFN) system comprises a series of antiviral IFN cytokines, classified as type I, II, and III based on their distinct molecular signatures and recognition receptors in cells, to induce hundreds of IFN-stimulated effector genes (ISGs), exerting various antiviral and other immunomodulatory functions (Figure 1) [16,17,18]. The IFN molecules of three IFN types are further designated into subtypes, which include the single IFN-γ for type II and IFN-λ1-4 for type III, such as in humans. There are multiple subtypes of type I IFNs, which include general subtypes of IFN-α and IFN-β produced by most cells, and more cell-specific subtypes, including IFN-ε (reproductive tract), IFN-κ (keratinocytes), IFN-ω (leukocytes/epithelial cells), and species-specific subtypes of IFN-δ (pigs), IFN-τ (cattle), and IFN-ξ (mice) [16,17,18]. 

Studies using transcriptomic analysis in SARS-CoV2-infected human bronchial cells or IFN assays in clinical plasma samples demonstrated a distinct immune-reaction phenotype in symptomatic COVID-19 patients, being a highly impaired interferon (IFN) response [19,20]. The impaired type I IFN response was characterized by decreased IFN-α/β expression in both SARS-CoV-2-infected human bronchial cells and circulating mononuclear blood cells, which was diagnosed together with persistent viremia and an exacerbated inflammatory response upon reactions to increased pro-inflammatory mediators, including tumor necrosis factor–α (TNF-α) and interleukin (IL)-6 [19,20]. Together with other previous in vitro studies, these data suggest that SARS-CoV-2 bears similar antagonistic mechanisms as other severe human coronaviruses (i.e., SARS and MERS) to interfere with the host IFN signaling, especially the production of type I IFNs (Figure 1) [21,22]. In contrast, other studies by Lee et al. (2020) and Lucas et al. (2020) detected that patients with severe COVID-19 had a sustained type I IFN response and consistent pro-inflammatory response in the blood of patients subjected to severe COVID-19 [23,24]. Contradictory results about type I IFN responses in COVID-19 patients may come from the disparity of criteria to define disease severity and different sampling times during the disease progression [25]. In addition, using large cohorts of COVID-19 patients in European countries, recent genome-wide association studies (GWAS) have significantly associated several critical genetic loci with severe COVID-19, which contain genetic regions spanning multiple genes that are centered in both chemokine and IFN signaling [26,27]. All these studies highlight the potential role of IFN signaling in determining the host susceptibility to SARS-CoV-2 infection and the progression of severe COVID-19 [19,20,21,22,23,24,25,26,27]. 

Interferon signaling, for either IFN induction or action, is not a linear cascade but an interacting network, dynamically adapting to alternative and crosstalk with other cytokine signaling pathways [16,17,18,25,27]. For IFN induction signaling during an RNA-virus infection as in COVID-19, the typical pathway is triggered by viral RNA through membrane-bound or cytoplasmic receptors (TLRs or RLR, as in Figure 1) and culminated at IFN-regulatory factor (IRF)-3/7 activation and IFN expression. Alternatively, animal cells are also capable of inducing IFN expression through cellular receptor-like cyclic GMP-AMP synthase (cGAS) to detect pathogenic DNA (pDNA) motifs from bacteria, viruses, and dead cells and to activate a stimulator of IFN genes (STING)-dependent pathway for IFN and inflammatory cytokine production (Figure 1, bottom-left panel). Similarly, for IFN action signaling, the canonical IFN signaling is through the engagement of membrane-bound IFN receptor (Figure 1, IFNA/LR for type I and III IFNs, respectively) and activation of STAT1/2 and ISGF3 transcription factors, leading to robust expression of hundreds of classical IFN-stimulated genes (ISGs, such as ISG15, MxA, IFITM, etc.), which exert antiviral role to restrict viral replication and spreading [16,17,18]. Alternatively, IFN signaling may divert to or synergize with TLR-mediated or cytokines (mainly TNF) signaling pathways to epigenetically promote the expression of a group of recently characterized non-canonical ISGs (non-ISGs) [18,28,29]. Two newly characterized non-canonical ISGs are inflammatory cytokine IL-6 and angiotensin-converting enzyme 2 (ACE2), a key component in the renin-angiotensin-aldosterone system (RAAS) and adopted by SARS-CoV-2 as a primary cellular receptor for infection [30,31,32]. For an RNA-virus infection like in COVID-19, the canonical IFN induction and action signaling are plausibly activated early to induce IFN and ISG production due to cell perceiving the presence of viral RNA in infected cells. The non-canonical IFN signaling for that responding to pDNA through cGAS-STING and non-canonical ISG stimulation via IFN-TNF epigenetic coordination might occur at the later stage, accompanying massive cell death from pyroptosis (a highly inflammatory form of programmed cell death in infected cells) and NETosis (an immunologically regulated form of neutrophil cell death), as seen in severe COVID-19 cases [16,17,18,33,34,35,36,37,38]. In addition to induction of IFNs/ISGs, the canonical and especially non-canonical IFN signaling pathway also lead to the production of inflammatory cytokines, which is further exacerbated by the virus suppression of ACE2 activity to develop into a cytokine release syndrome (CRS) or cytokine storm [30,31,34,35,36,37,38]. We propose that the integration of both canonical and non-canonical IFN signaling sufficiently addresses the contradictory observations from different studies, as discussed previously [19,20,21,22,23,24,25]. It explains that: (1) the weak IFN response is due to SARS-CoV-2-suppression on the canonical IFN signaling mainly triggered by viral RNA species, which signifies the early stage of the disease prior to severe progression [19,20,21]; (2) the robust IFN/ISG observations in severe COVID-19 cases accumulate consequential activation of non-canonical IFN signaling through both cGAS-STING for IFN production and IFN-TNF epigenetic regulation for ISG expression [23,24,33,34,35,36,37,38], which mostly happen at the late stage of the severe COVID-19 or when patients experience the complication of progressive pneumonia and multi-organ damage [23,24]. To support this proposal, the most known IFN antagonistic mechanisms of SARS-like coronavirus evolve to target major components of IFN canonical signaling, especially for IFN induction (Figure 1) [21]. Intensively, a study by Christopher et al. (2020) indicated that the IFN suppression of SARS-CoV-2 (probably through NSP3 on IRF3) effectively curated inflammatory responses through the cGAS-STING pathway, correlating to immunopathies from IFN dysregulation, which is worsen in severe COVID-19 [37,38,39]. 

## 2. Immunopathological Effect of Dysregulated IFN Responses

The suppression of IFN response, especially IFN production at the early stage of COVID-19 progression, diminishes the host capacity to restrict (thus benefits) the virus spreading [19,20,40]. Notably, the IFN system, like all other immune mechanisms, can be a double-edged sword to cause immunopathies, given it is not activated appropriately at the right time or intensity [41,42,43]. As in COVID-19, both the early stage of type I IFN deficiency and the late stage of IFN persistence could be a hallmark of severe COVID-19 [19,20,21,22,23,24]. As well studied in the cases of major autoimmune diseases and chronic viral infections, type I IFNs (IFN-α and IFN-β) are widely associated with immunopathology [33,40,41,42,43]. In contrast, type III IFN (IFN-λ) responses are restrictively mucosa-specific and exert antiviral defense with less damage from pro-inflammatory responses [17,43]. Accordingly, IFN-λ has been thought to have therapeutic advantages in COVID-19 [43]. However, updated studies in COVID-19 complicate the prophylactic promise of type III IFN-based clinical trials. Broggi et al. determined the subtype-dependent stimulation of type I and type III IFNs in the upper airway (naso-oropharyngeal swabs) and lung (BALF) samples and their correlation to COVID-19 patient morbidity [44]. Data showed that the virus-positive BALF samples from the severe COVID-19 patients in ICUs contained significant higher human IFN-α/β and type III IFN-λ2/3 but not IFN-λ1 compared with either the virus-positive or -negative swab samples [45]. Further data from in vivo mouse models indicate that the inductive expression of IFN-α/β and IFN-λ2/3 by the lung immune cells (primarily dendritic cells) causes damage to the lung epithelium, which hampers lung repair and increases susceptibility to lethal bacterial coinfections [44,45,46]. Indeed, a meta-analysis evaluated 4.3–9.5% of COVID-19 patients with a bacterial infection, which was more common in severe patients (8.1%) [47] and so were the incidences of co-infection from other microbes, including fungi and other viruses, in critically ill COVID-19 patients who suffer dysfunctional IFN and other immune reactions [48]. As mammalian IFN-α and IFN-λ2/3 subtypes evolve more inductive and antiviral activity than the epithelial-specific IFN subtypes (such as IFN-β and IFN-λ1) [49,50], the robust reaction of inflammatory IFN responses via recruited immune cells in the lung certainly deteriorate the pulmonary homeostasis maintained by the epithelial IFN subtypes, which is more constitutively expressed by pneumocytes prior to immunopathic IFN responses in severe COVID-19. Therefore, the more subtype-specific examination of the immunomodulatory and antiviral roles of both type I and type III IFNs in SARS-CoV-2 infection is imperative for IFN-based prophylactic development [25]. 

## 3. Evidence from Life-Threatening COVID-19 Cases with Inborn IFN Deficiency 

By genetic screening of 659 patients with life-threatening COVID-19 pneumonia, relative to 534 subjects with asymptomatic or benign infections, Zhang et al. (2020) detected an enrichment in a functional deficiency of 13 human gene loci that are known to govern TLR3- and IRF7-mediated antiviral IFN induction signaling in the severe COVID-19 patients [51]. These inborn errors in IFN induction ascribed to 23 patients (3.5%) who experienced life-threatening COVID-19 and aged 17 to 77 years. Despite a small proportion, the correlation indicated a group of the genetic extremity (compared with progressive IFN suppression by the virus and potential comorbidity conditions) in IFN deficiencies, underlying life-threatening COVID-19 patients without prior severe infection [51]. Another study by Bastard et al. (2020) revealed an autoimmune blocking of IFN action signaling [52]. In this case, they detected 101 of 987 (10.2%) patients with life-threatening COVID-19 pneumonia had auto-antibodies (auto-Abs), which were capable of binding and functionally blocking out almost all subtypes of type I IFNs, particularly of IFN-α, IFN-ω, and both IFN-α/ω subtypes, in further antiviral regulation [52]. In a few cases, the auto-antibodies were also detected against the tissue-specific type I IFN subtypes, including IFN-ε and IFN-κ typically expressed in the reproductive tract and skin keratinocytes, respectively [53,54]. In comparison, these auto-Abs were rarely found in the control cohort (663 individuals) who were SARS-CoV-2-positive but asymptomatic or with mild signs [52]. Comparably, auto-Abs against type I IFNs have been previously reported in patients subjected to IFN therapies and of systemic lupus erythematosus [55,56] and detected in almost all patients with autoimmune polyendocrinopathy syndrome type I (APS-1) [52,57]. In addition, 95% of the patients with the IFN auto-Abs have been male, which may at least partially explain why men face a higher risk of severe COVID-19, resulting in a higher risk of mortality [10,11,52]. Collectively, evidence from both inborn deficiency and auto-immune blocking of IFN function elegantly demonstrate that IFN signaling is a critical determinant of severe COVID-19 progression [51,52]. 

## 4. Category of IFN Dysregulation Underlying Severe COVID-19 Development 

**Figure 2:**
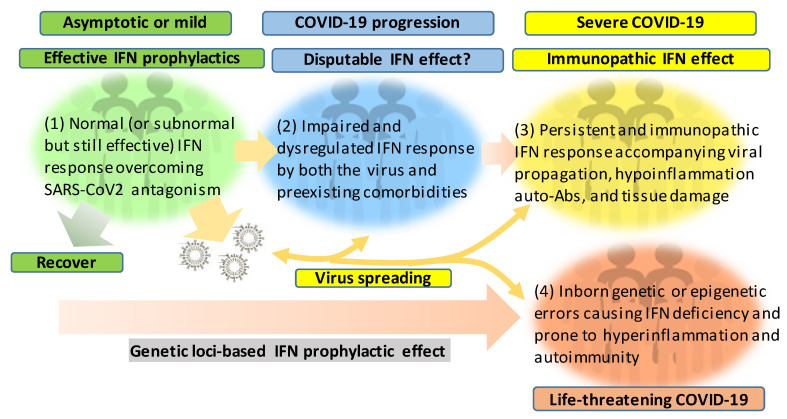
Schematic of patient cohorts of SARS-CoV-2 infections based on the severity of COVID-19 and underlying IFN responses. The effective or dysregulated interferon (IFN) response underlies the development of severe and life-threatening COVID-19. The dysregulation of IFN response can progressively result from the viral antagonism/virulence, preexisting comorbidities, gender/age inclination, and exacerbated hyper inflammation, with the extremal genetic flaws impairing the IFN signaling pathway. Hence, the prophylactic or therapeutic effect of IFN therapies should be designed and more dependent on the spatiotemporal kinetics of IFN responses during SARS-CoV-2 infection and the disease progression. In addition to its evolving antagonism to divert the host IFN response, the high contagiousness of SARS-CoV-2 also comes from the efficient virus infection and spreading by the non-hospitalized individuals who are asymptomatic or only have mild signs.

Figure 2 recaps our understanding of the dynamic interaction of the host IFN system with SARS-CoV-2 infection and the progression of COVID-19 into a severe status. The majority of healthy individuals, who are capable of mounting effective IFN responses during the early phase of the viral infection, will be recovered naturally or without intensive medical care to escape from the worse progression [58,59,60]. However, for another proportion of patients, who have pre-existing comorbidity or concur with a chronic inflammatory condition, their IFN response will be swayed to an immunopathic situation to exacerbate pneumonia in a severe COVID-19 development [61,62,63]. Dysregulation of IFNs and other immune factors have been associated with aging, sex difference, and pre-existing medical conditions, which have been clinically associated with a higher risk of severe COVID-19 [10,11,12,61,62,63]. Studies have shown that the capacity of both blood and lung dendritic cells (DCs), as a group of major IFN producers, in IFN production is severely impaired in aged individuals when compared to juveniles. On the contrary, blood DCs from aged people secrete higher basal levels of pro-inflammatory cytokines/chemokines, including IL-6, TNF-α, CXCL-8, CXCL-10 [64,65]. Together with other aging-associated lymphocytic abnormalities [66], this IFN and inflammatory dysregulation in DC response in aged individuals may invoke lung inflammation, impair antiviral resistance, and exaggerate major clinical signs as exacerbated in severe COVID-19 [8,9,10,11,12]. For the sex difference of IFN response, studies have demonstrated that plasmacytoid DCs (pDC) from healthy females are more potent to produce type I IFNs via TLR7-mediated signaling than the pDCs from males [67,68]. Plasmacytoid DCs serve as natural IFN producers and efficient sentinels in orchestrating antiviral immunity. This finding implicates an inferior status of males in the early antiviral IFN induction, a suitable stage for most IFN-based clinical trials having positive effects [25]. As for most preexisting medical conditions, including cardiovascular diseases, hypertension, obesity, and diabetes mellitus, which increase the risk of severe COVID-19 [61,63], many studies have unraveled the progressive incidence of IFN insensitivity and chronic inflammation and have been reviewed elsewhere [40,41,42,69,70,71]. In addition, a pathological consequence from persistent IFN and pro-inflammatory response, as well as the remarkable presence of auto-Abs, represent typical pathological mechanisms underlying most autoimmune diseases, including diabetes, multiple sclerosis, and systemic lupus erythematosus (SLE) [40,41,42,69,70,71]. The dysregulation of IFN and other immune factors in the COVID-19 patients with pre-existing comorbidities could be further complicated by the virus attacking endothelial cells to cause vasculitis, aneurysms, and coagulopathy, as well as tissue damage in the kidney, heart, and even brain [72,73,74,75]. The dysregulation of the IFN response can progressively result from the viral antagonism and virulence during viral replication (Figure 1). Furthermore, the preexisting comorbidities, gender and age inclination, and, particularly, exacerbated hyper inflammation associated with the IFN immunopathies and rigorous viral infection will undermine the distinctness of immune and pathological responses and lead to a life-threatening situation or death [10,11,12,61,62,63]. The inborn genetic and autoimmune deficiency of IFN response has been shown in about 14% of the examined life-threatening COVID-19 patients [51,52] who may experience sudden consequence even without a severe progression, thus further associating the dysfunction of IFN response with severe and life-threatening COVID-19 [51,52]. Hence, the prophylactic or therapeutic effect of IFN trial regimens should be carefully designed based on the temporal characteristics and subtype specificity of IFN responses during SARS-CoV-2 infection and the disease progression [25,49,50,53,54,76]. 

## 5. Conclusive Remarks: Precise IFN Response Kinetics and Application to COVID-19 Clinical Trials 

Effective IFN response or IFN dysregulation constitutes a key determinant of COVID-19 prognosis, which also highlights the potential of IFNs for therapeutic intervention [25]. Prophylactic administration of IFNs at the early stage prior to pneumonia progression may antagonize the viral suppression on IFN production and elicit an autonomous antiviral state in affected cells to block viral infection and COVID-19 pathogenesis. An early trial study (NCT04320238) showed that daily IFNα nasal drops enhanced the protection of at-risk healthcare workers from COVID-19 over 28 days without noticeable adverse effects [77]. However, the COVID-19 therapeutic effect of IFN treatments remains controversial, with respect to particularly the timing of administration and the pre-existing medical condition according to COVID-19 progression [25,78]. Interferon signaling has intricating crosstalk with multiple inflammatory cytokines, including TNF-α, IL-6, because they intersect in using some common intracellular signaling components [16,27]. In this context, the prophylactic effect of early IFN application may actually mitigate the CRS through the antiviral and anti-inflammatory effect of some epithelial-specific IFN subtypes. However, extensive validation of subtype-specific activity is warranted for better optimization of IFN’s clinical uses [79,80,81]. By contrast, clinical trials of relevant IL-6, TNF, and JAK STAT inhibitors and blocking antibodies are applied to the adverse side of dysregulated IFN response, which are devised to mitigate the pathological IFN and pro-inflammatory response sustained in severe COVID-19 [79,80,81]. Recent studies, per significant association of life-threatening COVID-19 with inborn genetic flaws and auto-Abs that block IFN response, genetically and epigenetically, reveal the critical role of IFN dysregulation in severe COVID-19 [51,52]. In most other severe COVID-19 patients without genetic errors in IFN-relevant gene loci, IFN dysregulation is progressively worsened and associated with the situation of pro-inflammation and immunopathy, which is prone to autoimmunity [41,61,62,63,82,83,84]. In addition, the high correlation of severe COVID-19 with seniority, males, and individuals with pre-existing comorbidities will be plausibly explained by the coincidence of IFN dysfunction in these listed situations, which have been reviewed elsewhere [41,82,83,84,85,86]. In addition, ACE2, a key enzyme of RAAS and sneaked as a primary receptor by SARS-CoV-2 infection, has been recently identified as a non-canonical ISG like IL-6 in response to IFN-induced epigenetic regulation [18,28,29,30,31,32]. Because the expression and affinity of ACE2 to SARS-CoV-2 determine host susceptibility and cell tropism [28,29,30,31,32], the dysregulated IFN response will further deteriorate the viral infection in multiple organs and incapacitate a series of functions regulated through the RAAS axis [30,86]. This will certainly complicate the understanding and application of IFNs, particularly for the treatment of severe COVID-19 [25,30,86]. All these call for a better understanding of the spatiotemporal characteristics and subtype-specificity of IFN response to SARS-CoV-2 infections, which are warranted to devise IFN-related prophylactics and therapies. It is noteworthy that all designed IFN therapies, which are based on normal IFN signaling, will be not properly functional in individuals who have an inborn genetic or auto-immune deficiency of the IFN system [52,53]. This will demand early diagnosis of this kind of genetic and auto-Ab errors in potential and hospitalized patients who are irresponsive to IFN-based treatments [27,52,53]. 

## Abbreviations

cGAS	cyclic GMP–AMP synthase
IFNA/LR	interferon-alpha/beta OR lambda receptor
IKKε	IκB kinase-ε
IRF	IFN regulatory factor
ISG	IFN-stimulated gene
JAK	Janus kinase
MAVS	mitochondrial antiviral signaling protein
ORF	open reading frame
P	phosphate
TLR/RLR	Toll-like receptor or retinoic acid-inducible gene 1-like receptors
SARS-CoV	severe acute respiratory syndrome coronavirus
STAT	signal transducer and activator of transcription
STING	signaling effector stimulator of interferon gene
TBK1	TANK-binding kinase 1
TRAF3	tumor necrosis factor receptor-associated factor 3
TYK2	tyrosine kinase 2
